# A Deep Learning Ensemble Approach for Automated COVID-19 Detection from Chest CT Images

**DOI:** 10.3390/jcm10245982

**Published:** 2021-12-20

**Authors:** Gaetano Zazzaro, Francesco Martone, Gianpaolo Romano, Luigi Pavone

**Affiliations:** 1 CIRA—Italian Aerospace Research Centre, 81043 Capua, Italy; G.Zazzaro@cira.it (G.Z.); F.Martone@cira.it (F.M.); G.Romano@cira.it (G.R.); 2 IRCCS Neuromed, 86077 Pozzilli, Italy

**Keywords:** COVID-19, computed tomography, medical imaging, deep learning, transfer learning

## Abstract

Background: The aim of this study was to evaluate the performance of an automated COVID-19 detection method based on a transfer learning technique that makes use of chest computed tomography (CT) images. Method: In this study, we used a publicly available multiclass CT scan dataset containing 4171 CT scans of 210 different patients. In particular, we extracted features from the CT images using a set of convolutional neural networks (CNNs) that had been pretrained on the ImageNet dataset as feature extractors, and we then selected a subset of these features using the Information Gain filter. The resulting feature vectors were then used to train a set of k Nearest Neighbors classifiers with 10-fold cross validation to assess the classification performance of the features that had been extracted by each CNN. Finally, a majority voting approach was used to classify each image into two different classes: COVID-19 and NO COVID-19. Results: A total of 414 images of the test set (10% of the complete dataset) were correctly classified, and only 4 were misclassified, yielding a final classification accuracy of 99.04%. Conclusions: The high performance that was achieved by the method could make it feasible option that could be used to assist radiologists in COVID-19 diagnosis through the use of CT images.

## 1. Introduction

In March 2020, the new coronavirus (COVID-19) pandemic was declared by the World Health Organization (WHO). As of now, there have been about 239 million confirmed cases of COVID-19, including more than 4 million deaths, according to the WHO [1]. The main common symptoms of COVID-19 include fever, dry cough, and tiredness. Since the virus may cause pneumonia as well as breathing difficulties or shortness of breath, chest pain or pressure, and loss of speech or movement in more aggressive infections, many healthcare systems around the world have suffered a breakdown, especially in terms of their intensive care units [2,3]. The gold standard for COVID-19 diagnosis is the nucleic acid kit for reverse transcription-polymerase chain reaction (RT-PCR) [4]. However, this method has several limitations, including false negatives (low sensitivity) [5,6], variability in diagnostic accuracy over the disease course [7], and a limited testing capacity in many countries [8]. Additionally, the ability of RT-PCR to detect COVID-19 strictly depends on the viral load. Medical imaging methods such as chest X-rays (CXR) and computer tomography (CT) can play a significant role in the diagnosis of COVID-19 [9,10], especially when they are used in combination with RT-PCR. In fact, they are very useful for monitoring disease progression and thus for optimizing the treatment strategy for the patient. CXR is a fast, cheap imaging method that is commonly used for the diagnosis of pneumonia worldwide [8,9]. It is less invasive than CT since it requires a lower dose of radiation. CXR is essential to evaluate pneumonia, pleural effusion, or pulmonary edema in COVID-19 patients, but its sensitivity in diagnosing COVID-19 is quite low [11]. CT is a medical imaging method that is based on X-rays and consists of many two-dimensional slices that allow high-resolution 3D images of the investigated body tract to be obtained through the application of a reconstruction algorithm. Chest CT is also widely used to diagnose COVID-19 because it seems to provide better diagnostic accuracy compared to CXR [12]. Although it is a new emerging disease, the intense research activity on the imaging data that can be obtained from COVID-19 patients that has been performed since the beginning of this pandemic has identified the imaging characteristics of COVID-19 [10]. CT findings include bilateral pulmonary parenchymal ground-glass and consolidative pulmonary opacities, sometimes with a rounded morphology. Furthermore, chest-CT scans have revealed incidental findings that are consistent with COVID-19, even in patients without respiratory symptoms [13]. The analysis of a CT scan requires an expert radiologist and takes about 10 min. This is a tedious and repetitive task that may cause doctors to experience mental fatigue, and it does not allow fast detection or screening in large-scale investigations. Furthermore, it has been demonstrated that the interpretation of CT images for COVID-19 diagnosis that have been made by radiologists do not have high sensitivity [14]. For these reasons, novel approaches have been proposed in order to find automated methods to detect COVID-19 in CT images. All of these approaches use artificial intelligence (AI) techniques, particularly those that are derived from machine learning (ML), which are considered to be a prominent tool for the prediction and diagnosis of numerous diseases [15]. In recent years, many research groups have tried to address the need for automated COVID-19 detection by proposing machine learning approaches that are based on clinical neuroimaging data. Although there are many studies that make use of CXR [16,17,18] or both image types (CXR and CT) [19,20], we only reported studies that make use of chest CT images, because these images are more accurate in COVID-19 diagnosis [12]. Shan et al. [21] proposed a deep learning-based segmentation system for quantitative infection assessment. The method includes the auto contouring of infection regions and the estimation of shape, volume, and percentage of infection, achieving Dice similarity coefficients of 91.6 ± 10.0% between automatic and manual segmentations, demonstrating a dramatic reduction in the time needed to delineate the infection compared to the manual approach. Alshazly et al. [22] used a number of different deep network architecture with transfer learning and conducted extensive sets of experiments to optimize the performance of the models on two different CT image datasets; the best model achieved an average accuracy of 99.4% and 92.9% on the two datasets. Xu et al. [23] used multiple CNN models to classify CT image datasets in two classes, namely COVID-19 and Influenza-A viral pneumonia, and to calculate the infection probability of COVID-19, achieving an overall accuracy of 86.7%. Wang et al. [24] proposed a deep learning algorithm using CT images to screen for COVID-19 during the influenza season using a transfer learning neural network that was based on the Inception network. Gozes et al. [25] presented a system that utilized both 2D and 3D deep learning models, modifying and adapting existing AI models to classify coronavirus and non-coronavirus cases. These authors achieved classification results of 0.996 AUC (Area Under the ROC Curve), with a sensitivity ranging from 96.4% to 98.2% and specificity from 92.2% to 98%. Hasan et al. [26] proposed a DenseNet-121 Convolutional Neural Network to classify and identify COVID-19 patients from CT images, achieving an accuracy of 92% with 95% recall. Rohila et al. [27] presented a method that could be used to detect varying degrees of COVID-19 infection from full chest CT scans by using a deep CNN model with ResNet-101, achieving an overall accuracy of 94.9%. Soares et al. [28] proposed an eXplainable Deep Learning approach (xDNN) that used a dataset that contained 2482 CT scans in total, 1252 CT scans that were positive for SARS-CoV-2 infection, and 1230 CT scans for patients who were not infected with SARS-CoV-2, achieving an F1 score of 97.31%. Loddo et al. [29] presented a method in which they first compared different architectures on a public and extended reference dataset to find the most suitable one, and then proposed a patient-oriented investigation to determine which network had the best performance. Finally, they evaluated their robustness in a real-world scenario, which was represented by cross-dataset experiments. They achieved 98.87% accuracy in their network comparison, 95.91% accuracy for patient status classification, and 70.15% accuracy in the real-world scenario. In this paper, we present a method that makes use of chest CT images taken from a publicly available dataset [28] in order to conduct an automated classification of the images in two classes, namely COVID-19 and NO_COVID-19. The method consists of the following steps:

1. A feature extraction step using a set of convolutional neural networks that have been pretrained on the ImageNet dataset [30]. ImageNet is a large, publicly available database of natural images with 1000 object classes that was specifically created for computer vision and, more recently, has been widely used for deep learning and transfer learning research. There are more than 1.2 million training images, 50,000 validation images, and 100,000 test images that are available in the database with relative annotations. The images are mostly in the JPG file format and vary in size.
2. A feature selection step that uses the information gain filter.
3. Training of the generated models using machine learning approaches.
4. A model selection step.
5. Classification of CT images into one of two classes using a majority voting approach.

## 2. Materials and Methods

### 2.1. Dataset

In this study, we used a publicly available multiclass CT scan dataset [28] that contained 4171 CT scans of 210 different patients, out of which 2167 correspond to 80 patients who were infected with SARS-CoV-2, a diagnosis that had been confirmed by RT-PCR. These data were collected at the Public Hospital of the Government Employees of Sao Paulo (HSPM) and the Metropolitan Hospital of Lapa, both in Sao Paulo, Brazil. The dataset is composed of CT scans in the png format that have been divided into 757 CT scans from healthy patients (15 CT scans per patient on average), 2167 CT scans from patients infected by SASR-CoV-2 (27 CT scans per patient on average), and 1247 CT scans from patients with other pulmonary directions (16 CT scans per patient on average). As our goal was to develop a method that would be able to distinguish CT images of COVID-19 patients from those of NO_COVID-19 patients, we decided to gather CT scans of healthy patients and of patients with other pulmonary diseases together in one group in order to simplify the training step. As a result, we created a balanced dataset that composed of 2167 CT scans with the COVID-19 label and 2004 CT scans with the NO_COVID-19 label.

### 2.2. Proposed Approach

The analysis of the whole dataset with all the 4171 CT images began with the separate use of N Deep Neural Architectures that had been pretrained on the ImageNet dataset, which is freely available through the TensorFlow framework [31], with the main goal of extracting different N sets of features. We chose to use the ImageNet dataset and transfer learning due to their increasing use in deep learning research and in many papers describing automated COVID-19 detection methods [25,28]. Once the N sets of features had been obtained, we performed a feature selection step, which was useful to reduce the dimensions of the dataset, by using the information gain filter [32], which is based on information entropy, which is widely used in ML. This filter will be described in detail in Section 3.1.

**Figure 1:**
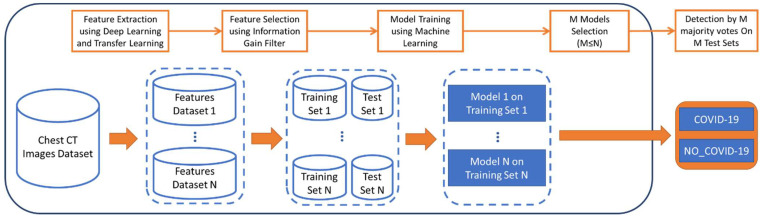
Summary of the pipeline used in this study.

Then, a model was induced, where 90% of each set of features was used for the training set; its performances, e.g., its accuracy, was evaluated by applying the cross-validation method [32] with 10 folds, which allows models to be trained and evaluated when using small datasets. The remaining 10% of the examples was used for the final evaluation. In order to select the best deep neural architectures, the k-nearest neighbors (k = 1) (k-NN) algorithm was applied to the different N datasets of features by using the 10-fold cross validation technique. The M (M ≤ N) selected k-NN results were thus used for the final tests on the M Test Sets. Finally, the M k-NN classifications were included in an ensemble for a majority vote, and the performances of a set of independent images (the remaining 10% of the whole dataset) was presented for final testing; in particular, each image of the test set was classified according to the M k-NN, and it was assigned to the cored class (COVID-19 or NO_COVID-19) by majority. A summary of the steps that were used in our method is shown in Figure 1.

### 2.3. Transfer Learning

Transfer learning (TL), also called inductive transfer, knowledge transfer, or learning to learn [33], is an ML method that allows the domains, tasks, and/or data distributions that are used in training and testing phases to be differentiated. TL is a method that utilizes the knowledge that is achieved by a learning model after considering a specific problem to solve a distinct but similar task [34]. This transferred knowledge can be applied to a new dataset, the size of which is usually insufficient to train a new accurate model from scratch. Some authors [35] have defined three main different TL sub fields that are based on different conditions among the sources and the target domains and tasks, as reported in Table 1.

TL approaches can be divided into four main groups [33]:

1. Instance-based: Mainly refers to instance weighting strategy.
2. Feature-based: Transforms the original features to create a new feature representation.
3. Parameter-based: Transfers the knowledge at the model/parameter level.
4. Relational-based: Focuses on the problems in relational domains. This approach transfers the logical relationship or rules learned in the source domain to the target domain.

TL has been applied in many domains and application fields for many tasks. In recent studies, TL has been used in combination with the use of deep learning [36], for example, by means of convolutional networks (CNNs), where the method needs an initial training of a net for a given task using a large dataset. For example, in [17], the authors proposed an application of a TL method in the medical field that was aimed at the automatic detection of a COVID-19 infection from chest X-ray images. Since few chest images from infected patients were publicly available, the authors used different architectures of CNNs to apply a TL method.

The architectures of the networks were trained on ImageNet [30], and after being adapted to be feature extractors for the chest X-ray images, the proposed method achieved an accuracy of 98.5% by combining the extracted features with a classifier based on a support vector machine algorithm. This approach is called feature extraction for TL [37], where the extracted features are used in a new model later on that will process its classification. As such, the accessibility of a considerable set of data is the main factor that can guarantee the success of the training phase, and the use of large datasets for the initial training of the neural networks enables high performance in smaller or poorer datasets. Moreover, the feature extraction that is achieved with TL allows a large number of features to be extracted by generalizing the problem and by avoiding excessive customizations and adjustments.

### 2.4. Convolutional Neural Networks (CNNs)

**Figure 2:**
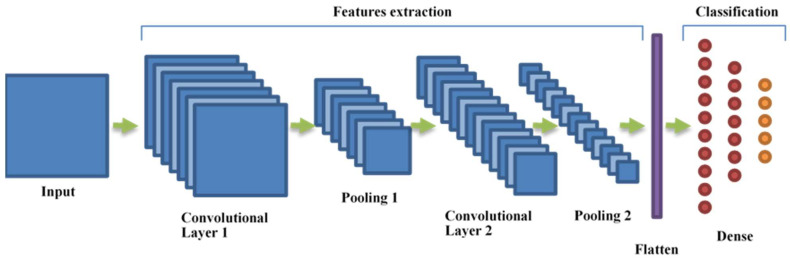
The typical architecture of a convolutional neural network.

Currently, CNNs have achieved state-of-the-art performance in computer vision tasks such as object recognition, image classification, and image segmentation. In these networks, the analysis of the input image proceeds through several layers of convolutional filters. The elements of the matrices that are implementing these filters are calculated during the training process, maximizing the performance of the network on the training dataset. In this adaptation lies the strength and flexibility of convolutional networks. A convolutional layer extracts those characteristic elements, called features, which are useful in the analysis process, from its input. A layer of the network implements many different convolutional filters and collects them into a so-called feature map. A convolutional layer may be followed by a pooling layer: a window of a predetermined size is scrolled along the layer output, and the values that are contained in it are replaced by their average (average pooling) or by the maximum element (max pooling). The pooling layers make the network more robust with respect to translations and reduce its output size. After the last convolutional or pooling layer, the feature maps are collected into a single one-dimensional vector of features that can be used as a unique descriptor of the content of the entire image. This vector is then used as the input of a classical, fully connected neural network that can be used for classification. The typical architecture of a CNN is showed in Figure 2.

Varying the number of convolutional and pooling layers, the number of filters in the feature maps and the way in which the layers are connected results in a wide variety of CNN architectures [38,39,40,41,42,43,44,45].

In general, training a CNN from scratch requires a large number of labelled images that can be used for training that may not be available for the problem at hand. This limitation can be at least partially overcome by using a network that has already been trained on a large dataset and that has been deprived of the final classification layers as a feature extractor (transfer learning).

In this work, we use a number of different CNN architectures that have been pre-trained as features extractors on the ImageNet dataset for the classification of CT scans in COVID and non-COVID classes. The dataset that we used contains images of different sizes. This is not a problem for convolutional and pooling layers, but it does produce vectors of features with different lengths as the output. Since classification algorithms expect the input vectors to be of a fixed size, we added a global max-pooling layer to every CNN that we used after removing the classification layers, which gives the max output values of the feature maps in the last layer of the convolutional network, fixing the size of the output vectors of the features.

### 2.5. K-Nearest Neighbor and Majority Voting Approach

The classifications were compared, and a majority voting rule was defined for the assignment of the final class. Therefore, instead of running the risk of picking an unsuitable or not very accurate classifier, an ensemble model can be used to achieve “better” results. The idea is that no single model or criterion can truly capture the optimal classification (or class separation rules), but a collective of models can provide a more robust final classification. As is often quoted in the literature [32,46,47], a classifier that is based on the majority voting method often achieves better performance than the performance of a single component method.

In this work, the classifiers (or the ensemble components) were trained using the k-NN algorithm, fixing k = 1, by considering different datasets of extracted features. For the sake of clarity, k-NN is instance-based or a lazy learner, and it does not require model training: all pf the samples that belong to the training set are memorized, and all of them are considered to classify every test sample, computing the distances individually between the test and all of the training samples. For this reason, k-NN is also classified as a memory-based learner, and the procedure does not gain a classifier as output. In more detail, it only starts working during the testing phase to compare the given test observations with the nearest training observations. The k-NN algorithm is one of the most commonly used methods in data mining (DM) due to its simplicity and high performance in many applications. It has gained popularity through the work of Aha [48]. k-NN is widely used in many ML and DM tasks, such as classification, motif discovery, and anomaly detection. It has shown excellent results in several application domains and in a large number of classification problems, including satellite image scenes, handwritten digits, ECG patterns, web search, spell checking, and fraud detection. It is often successful cases where the decision boundaries are very irregular [49]. The algorithm represents a classification method that is based on learning by analogy, in which a new object is labeled based on its closest (k) neighboring objects (points). It is based on the simple assumption that similar inputs are usually related to similar outputs. In other words, in the simplest case where k = 1, the class of the instance that is most similar, or close, to the new vector is used as the output class. If k > 1 and k is odd, then the output class is assigned to the new instance by considering the majority of the k class of the k nearest instances. The algorithm computes the distances between each point in the test set and all of the points of the training set in order to achieve its nearest-neighbor list. “Analogy” (or “closeness”, or “nearness”) is usually defined in terms of Euclidean distance, but other choices are possible [50].

## 3. Results

### 3.1. Feature Extraction and Selection

The analysis of the whole dataset containing the 4171 CT images begins with the separate use of 26 deep neural architectures, which are listed in Table 2, with the main goal of extracting 26 different sets of features. Once we obtained these 26 sets of features, we performed a feature selection step, which was useful to reduce the dimensions of the datasets. This was performed using the information gain (IG) filter, which evaluates the worth of an attribute by measuring the information gain with respect to the class. We chose all of the features X for which IG(X) > 0. The IG can be calculated by the following formula [32]:

IG(Class,Attribute) = H(Class) − H(Class|Attribute)

where H is the information entropy (or Shannon entropy), which can be calculated with the following formula:

$$H\left(X\right)=-\overset{n}{\underset{i=1}{\sum }}p\left(x_{i}\right)\log\left(p\left(x_{i}\right)\right)$$

The minimum value of IG for each dataset was equal to 0. The reduction in the number of features varied from 6.17% for EfficientNetB0 to 61.1% for ResNet101V2. Table 2 summarizes the number of features that was selected by applying the IG for each deep neural network (and therefore for each dataset).

### 3.2. Model Training

The hold-out method was applied. In this strategy, each of 26 feature datasets with labeled examples was partitioned into two disjoint subsets, called the training set and the test set. A model was induced from each training set, which comprised 90% of the whole dataset; its performances, e.g., its accuracy, which is the ratio between the number of correctly classified instances and the total amount of instances, was evaluated by applying the cross-validation method with 10 folds, which allows models with small datasets to be evaluated. The remaining 10% of the examples was used for the final evaluation. For the sake of clarity, the test sets were made up of feature vectors that resulted from the same set of images. In order to select the best deep neural architectures, the k-NN (fixing k = 1) algorithm was applied for the training phase on the 26 different datasets of features by using the 10-fold cross validation technique.

The average accuracies of the 26 classifiers are showed in Table 3.

### 3.3. Model Selection

The k-NN algorithm was used also to select the best M (M ≤ N) neural architectures or, equivalently, the M datasets of the features. Thus, the M datasets were selected through the use of an arbitrarily selected accuracy threshold of 80% of correctly classified instances. As such, the k-NN algorithm was applied with a dual purpose: to obtain the M classification rules using the concept of proximity and also to select the best M neural architectures and therefore the M datasets of features. Only one of the neural architectures (InceptionResNetV2) among those that were considered did not meet the chosen criterion (accuracy threshold of 80%). The accuracy of the remaining 25 classifiers on the independent test set (10% of the whole dataset of CT images) varied from 81.5789% to 96.6507%, with an average accuracy of 91.5502%. These 25 k-NNs were used for final tests on the 25 test sets.

### 3.4. Classification

Finally, the 25 k-NN classifiers were included in an ensemble for a majority vote, and the performances of a set of 414 independent images (the remaining 10% of the whole dataset) were presented for final testing; in particular, each image of the test set was classified by each of the 25 k-NNs, and its final class (COVID-19 or NO_COVID-19) was assigned by majority. Considering the 25 k-NNs as components of an ensemble classification model and by assigning the class label with the majority voting method, we obtained a meta-classifier (ensemble classifier) of 99.0431% accuracy, a value that was higher than those of the accuracies of the singular components (Table 3). In particular, 414 images from the test set (10% of the complete dataset) were correctly classified, and only 4 were misclassified. In particular, the final model showed two false positives (two false alarms or two CT scans incorrectly classified as containing COVID-19 infection) and two false negatives (two CT scans incorrectly classified as not containing COVID-19 infection). The confusion matrix of the final ensemble model is shown in Table 4, where TP is the number of true positives, FP is the number of false positives, FN is the number of false negatives and, finally, TN is the number of true negatives. As reported in Table 3 and Table 4, the ensemble meta-classifier produces better results than the individual classification components do. Many performance metrics [32] can be calculated from the confusion matrix. The most common as well as the most useful for model comparisons are shown in Table 5, where the values of our meta-classifier are also reported.

## 4. Discussion

The described study presents an automated method that can be used to detect COVID-19 infections from chest CT scans by using deep learning-based approaches. Specifically, we used 26 pre-trained deep neural architectures for feature extractions, an information gain filter to select a subset of the previously extracted features for each dataset, and the k nearest neighbors (k = 1) algorithm for target class detection and model comparison and selection. A total of 25 of the 26 models achieved the arbitrarily determined accuracy threshold for correctly classified instances of 80%. The selected 25 k nearest neighbors classifications were also used as the ensemble components for a majority voting approach that was able to classify each input image into two different classes: COVID-19 and NO_COVID-19. Although the dataset contained three different classes of subjects (COVID-19, healthy, and subjects with other pulmonary diseases), we decided to group the images of healthy subject and those of patients with other pulmonary diseases into one class (NO_COVID-19) in order to have a balanced dataset. Thus, we did not test the ability of this method to discriminate between COVID-19 and other pulmonary disease. We achieved an ensemble classification accuracy of 99.04%, which is greater than the accuracies of each of the models 25 individual components. Moreover, the results that were achieved by the proposed method (Table 5) exceed the results of all of the other works that make use of chest CT images. In particular, our technique appears to be better since it exhibits a very low percentage of misclassified images (see Table 6).

Our approach is different with respect to other similar approaches since we used CNN models that had been pretrained using the ImageNet dataset alone and without any fine tuning and simple KNN models in an ensemble, which greatly reduced the computational burden of the training phase, which is a critical parameter in a clinical setting. In fact, the time needed for the analysis of a single CT image was about 19 s on average, with a minimum of 8 s for the smallest images to a maximum of 28 s for the largest. The CNNs that were used were implemented using the TensorFlow framework on a laptop with Intel(R) Core (TM) i7-8665U CPU, 16 GB RAM, with no discrete graphic card. Considering the very high performance that was achieved by our method and the very low computational times, we strong believe that with a few improvements, it could provide a reliable and accurate method that could help radiologists and clinicians during the diagnostic process. First, our method does not perform any kind of pre-processing on the input images, and this could affect the performance of the method because it is well-known that it is important to pre-process imaging data in order for the model to provide more efficient analysis and better consistency. Furthermore, the proposed method did not perform any fine tuning, which could greatly improve the performance of the model; thus, in future developments of our methodology, we will include an ad hoc fine-tuning feature for all or for a subset of the considered neural architectures. We did not perform any fine tuning in order to reduce the computational time that was required to make a decision because our goal was to search for a method with a good trade-off between performance and computational burden. Since our method is useful for the selection of the most promising neural architectures that are able to detect COVID-19 infection, we plan to only consider the neural architectures with the highest performances in future work in order to apply a fine-tuning step, which would also serve to evaluate the use of a different machine learning algorithm, instead of the KNN approach. In our work, the ensemble of pre-trained neural architectures showed better performances (Table 4 and Table 5) than those of the singular components (Table 3). This result could suggest that the high performances that were achieved by our methodology could also be due to the choices that were made via majority voting; moreover, this approach also differentiates our method from those that were examined in related works (Table 6). Finally, the dataset that was used in this study is not big since it contains about 4000 CT images, and the number of the patients to which such images belong to is limited. Increasing the amount of data would improve any deep learning model. Thus, in the future, we plan to test our method in larger COVID-19 research databases acquired from different sources in order to improve the performance of the model and to make the methods more generalized. In fact, for the feature extraction step, we used 26 deep neural architectures that had been trained on the ImageNet dataset. Since this dataset is not specific for the diagnosis of COVID-19, we think that training these architectures on a large dataset that is specifically designed for COVID-19 diagnosis would improve the performance of our method. Finally, the lack of clinical data that are associated with the images did not allow us to investigate the effects of the clinical characteristics of the patients on the performance of the method, and we plan to carry out such an investigation in future studies.

## Figures and Tables

**Table 1:** Transfer learning subsettings.

	Subsetting Name	Description	Label Information
1	Inductive TL	the target task is different, but related, from the source task	comes from the target domain
2	Transductive TL	the source and target tasks are the same, while the source and target domains are different	comes from the source domain
3	Unsupervised TL	the target is different from, but related to, the source task, and the focus is on solving unsupervised learning tasks in the target domain	is always unknown for both the source and the target domains

**Table 2:** Number of selected features by information gain for each deep neural network.

N	Deep Neural Network	Maximum Value of Information Gain	N. of Original Features	N. of Selected Features	Percentage Reduction of Features by IG
1	DenseNet121	0.145	1025	930	9.27%
2	DenseNet169	0.162	1665	1488	10.63%
3	DenseNet201	0.165	1921	1669	13.12%
4	EfficientNetB0	0.203	1281	1159	9.52%
5	EfficientNetB1	0.160	1281	1202	6.17%
6	EfficientNetB2	0.187	1409	1314	6.74%
7	EfficientNetB3	0.207	1537	1431	6.90%
8	EfficientNetB4	0.141	1793	1598	10.88%
9	EfficientNetB5	0.164	2049	1903	7.13%
10	EfficientNetB6	0.181	2305	2074	10.02%
11	EfficientNetB7	0.157	2561	2315	9.61%
12	InceptionResNetV2	0.126	1737	1533	11.74%
13	InceptionV3	0.186	2049	1899	7.32%
14	MobileNet	0.175	1025	829	19.12%
15	MobileNetV2	0.144	1281	889	30.60%
16	MobileNetV3Large	0.116	1281	1080	15.69%
17	MobileNetV3Small	0.111	1025	849	17.17%
18	ResNet50	0.333	2049	1735	15.32%
19	ResNet50V2	0.150	2049	955	53.39%
20	ResNet101	0.204	2049	1717	16.20%
21	ResNet101V2	0.183	2049	797	61.10%
22	ResNet152	0.284	2049	1665	18.74%
23	ResNet152V2	0.206	2049	1518	25.92%
24	VGG16	0.156	513	404	21.25%
25	VGG19	0.184	513	440	14.23%
26	Xception	0.138	2049	1071	47.73%

**Table 3:** Average accuracies of the 26 classifiers.

		Average Accuracy = 90.4548%	Average Accuracy = 91.5502% (without InceptionResNetV2)
N	Name	Accuracy of k-NN (k = 1) 10-Fold Cross Validated	Accuracy of k-NN (k = 1) on 10% Test Set
1	DenseNet121	93.4186%	93.3014%
2	DenseNet169	92.5926%	94.7368%
3	DenseNet201	91.3403%	90.1914%
4	EfficientNetB0	95.2038%	95.933%
5	EfficientNetB1	96.7493%	96.6507%
6	EfficientNetB2	93.6318%	94.7368%
7	EfficientNetB3	93.7393%	96.1722%
8	EfficientNetB4	92.8058%	93.5407%
9	EfficientNetB5	91.8732%	91.3876%
10	EfficientNetB6	88.5159%	87.5598%
11	EfficientNetB7	92.3261%	94.0191%
12	InceptionResNetV2	78.737%	77.2727%
13	InceptionV3	88.5425%	88.756%
14	MobileNet	90.9406%	90.6699%
15	MobileNetV2	91.3669%	93.7799%
16	MobileNetV3Large	88.5425%	90.1914%
17	MobileNetV3Small	84.3858%	83.7321%
18	ResNet50	95.3637%	96.1722%
19	ResNet50V2	81.1617%	81.5789%
20	ResNet101	94.1913%	93.5407%
21	ResNet101V2	86.2776%	84.9282%
22	ResNet152	94.1114%	96.89%
23	ResNet152V2	84.0927%	85.6459%
24	VGG16	94.7509%	93.3014%
25	VGG19	94.5377%	95.933%
26	Xception	82.6272%	85.4067%

**Table 4:** Confusion matrix on test set.

	YES	NO	Classified as
YES	TP = 215	FN = 2	
NO	FP = 2	TN = 199	
Meta-Classifier Accuracy 99.04%			

**Table 5:** Performance metrics of the meta-classifier on test set.

Symbol	Performance Metric	Definition as	What Does It Measure?	Value
CCR	Correctly Classified instance Rate—Accuracy	(TP + TN)/(TP + TN + FP + FN)	How good the model is at correctly predicting both positive and negative cases	0.9904
TPR	True Positive Rate—Sensitivity—Recall	TP/(TP + FN)	How good the model is at correctly predicting positive cases	0.9908
FPR	False Positive Rate—Fall-out	FP/(FP + TN)	Proportion of incorrectly classified negative cases	0.010
PPV	Positive Predictive Value—Precision	TP/(TP + FP)	Proportion of correctly classified positive cases out of total positive predictions	0.9908
AUC	ROC Area	Area under the ROC curve	Area under plot of TPR against FPR	0.997

**Table 6:** Related works for COVID-19 infection detection.

Author	ML Approach	Data Source	Transfer Learning	Achieved Performance
Alshazly et al. [22]	Pre-trained SqueezeNet, Inception, ResNet, ResNeXt, Xception, ShuffleNet and DenseNet CNN with fine tuning	2482 CT images + 746 CT images	Not Declared	Accuracy: 99.4% and 92.9% on the two datasets
Xu et al. [23]	ROI segmentation with 3D CNN + Classification with ad hoc ResNet-18 CNN	618 CT images	No	Accuracy: 86.7%
Wang et al. [24]	Pre-trained Inception CNN with fine tuning	1065 CT images	Not Declared	Accuracy: 79.3% Recall: 83% Specificity: 67% AUC: 0.81
Gozes et al. [25]	Pre-trained ResNet-50 CNN with fine tuning	206 patients CT scans	ImageNet	AUC: 0.996
Hasan et al. [26]	DenseNet-121 CNN	2482 CT images	No	Accuracy: 92% Recall: 95%
Rohila et al. [27]	Ad hoc deep learning network based on ResNet-101	1110 patients CT scans	Yes, but no ImageNet	Accuracy: 94.9%
Soares et al. [28]	xDNN (eXplainable Deep Neural Network)	2482 CT images	ImageNet	Accuracy: 97.4% Recall: 95.53% Precision: 99.16% AUC: 0.9736
Loddo et al. [29]	Pre-trained AlexNet, Residual Networks, ResNet18, ResNet50, ResNet101, GoogLeNet, ShuffleNet, MobileNetV2, InceptionV3, VGG16 and VGG19	470 + 194,122 Chest CT images	No	Accuracy: 98.87% (nets comparison) 95.91% (patient status classification)
Our approach	Pre-trained CNNs, k Nearest Neighbors with 10-fold cross validation, majority voting approach	2482 CT images	Yes ImageNet	Accuracy: 99.04% Recall: 99.08% Precision: 99.08% AUC: 0.997

## Data Availability

The CT data used in this study are publicly available, while the data coming from deep learning analysis are available upon request by contacting the corresponding author.
